# Ternary Sulfur/Polyacrylonitrile/SiO_2_ Composite Cathodes for High-Performance Sulfur/Lithium Ion Full Batteries

**DOI:** 10.3390/polym10080930

**Published:** 2018-08-20

**Authors:** Yusen He, Zhenzhen Shan, Taizhe Tan, Zhihong Chen, Yongguang Zhang

**Affiliations:** 1 School of Materials Science and Engineering, Hebei University of Technology, Tianjin 300130, China; hyshebut@163.com (Y.H.); shanzhenzhenhebut@163.com (Z.S.); 2 Synergy Innovation Institute of GDUT, Heyuan 517000, China; taizhetan@gdut.edu.cn; 3 Shenyang Institute of Automation, Chinese Academy of Sciences, Guangzhou 511458, China

**Keywords:** sulfur/lithium-ion full battery, prelithiated graphite anode, stabilized lithium metal powder, ternary sulfur/polyacrylonitrile/SiO_2_ composite, electrochemical performance

## Abstract

In the present study, a novel sulfur/lithium-ion full battery was assembled while using ternary sulfur/polyacrylonitrile/SiO_2_ (S/PAN/SiO_2_) composite as the cathode and prelithiated graphite as the anode. For anode, Stabilized Lithium Metal Powder (SLMP) was successfully transformed into lithiated graphite anode. For cathode, scanning electron microscopy (SEM) and transmission electron microscopy (TEM) revealed that SiO_2_ was uniformly distributed on S/PAN composites, where SiO_2_ served as an effective additive due to its ultra high absorb ability and enhanced ability in trapping soluble polysulfide. The tested half-cell based on S/PAN/SiO_2_ composite revealed high discharge capacity of 1106 mAh g^−1^ after 100 cycles at 0.2 C. The full cell based on prelithiated graphite//S/PAN/SiO_2_ composite system delivered a specific capacity of 810 mAh g^−1^ over 100 cycles.

## 1. Introduction

Battery storage systems have been widely used in various applications, such as wind and solar energy storage, emergency back-up power, peak shaving, load-leveling, and transportation electrification [1,2]. Lithium-ion secondary batteries are widely used in mobile communications, notebook computers [3,4], digital cameras, and other small electronic devices due to several interesting features [5], including high energy storage density, long service life, and elevated rated voltage [6]. However, the energy densities of lithium-ion batteries do not currently meet the demand, hence the development of new energy storage systems becomes important [7,8,9,10,11,12]. Lithium/sulfur (Li/S) batteries have high theoretical energy densities reaching up 2600 Wh kg^−1^, making them the most promising energy chemical power systems. In regard to this, elemental sulfur as cathode has low cost when combined with its environmental friendliness and high theoretical specific capacity (1672 mAh g^−1^) [13,14,15,16,17,18]. However, despite these criteria, the development of Li/S batteries still faces numerous challenges. Elemental sulfur is electrically insulating and the polysulfide generated during charging and discharging processes (Li+S_8_→Li_2_S_x_ (4 ≤ x ≤ 8)) is highly soluble in electrolytes [19,20].

The above issues could be solved by adding various types of conductive carbon materials [21,22,23]. Also, some polymer and oxide additives might enhance the structural stability of composites and improve the conductivity of matrix materials. On the other hand, it is important to suppress the “shuttle effect” of polysulfides [24].

Among composites, sulfur/polyacrylonitrile (S/PAN) showed high sulfur utilization with elevated initial capacity [21,25]. However, the poor electrical conductivities of S/PAN binary composites render the cycling and rate performances hindered. To this end, the Li/S battery exhibits enhanced electrochemical performance due to the different types of additives in the sulfur cathode, which results in a change in morphology and/or absorbing properties. The additive has the advantages of small dose and remarkable effect. Therefore, the use of additives in Li/S cells to change the morphology of S composite is one of the effective methods to improve the battery performance [26,27].

A method of containment at the cathode is to provide additives in the cathode matrix that can attract and hold polysulfides so that it does not diffuse to the negative electrode. To employ this concept [28], our previous study showed that the morphology of S/PAN composite greatly changed by adding small amounts of additives. Also, the composition morphology has been transferred from smooth to rough, effectively improving the electrochemical reaction at the electrode. In this work, we successfully synthesized S/PAN/SiO_2_ composites as cathode materials by wet ball milling, followed by heat treatment. The addition of small amounts of SiO_2_ nanoparticles was found to be beneficial for optimizing the surface morphology and favoring the homogeneous distribution of individual components. The above studies were based on traditional Li/S battery system, employing lithium metal foil as anode characterized by safety hazards, possible dendrite formation, short-circuiting, and cell thermal runway [29].

To improve the safety concerns of large-scale production of lithium/sulfur batteries, one promising strategy is to develop pre-lithiated commercial graphite anodes while using stabilized lithium metal powders (SLMP). In previous studies, some studies investigated the potential use of SLMP for overcoming the irreversible capacities of various anode systems [30,31]. Herein, we developed a novel sulfur/lithium-ion battery with pre-lithiated graphite anode, and the performances of the resulting pre-lithiated graphite//S/PAN/SiO_2_ composite battery were discussed.

## 2. Materials and Methods

The ternary cathode materials were prepared by first mixing 8 g sulfur (Shanghai Huzheng Nano Technology Co., Ltd., Shanghai, China), 2 g polyacrylonitrile (PAN) (Sigma-Aldrich, Shanghai, China), and 0.5 g SiO_2_. Anhydrous ethanol (Aladdin, Shanghai, China) was then added to the mixture for wet ball milling at 600 rpm and 2 h. Next, the obtained ball-milled product was dried and heat-treated under protective N_2_ atmosphere at 450 °C for 6 h. For comparison, S/PAN binary composite was also prepared while using the same experimental conditions.

The samples were characterized by X-ray diffraction (XRD, Bruker D8, Bruker, Karlsruhe, Germany), scanning electron microscopy (SEM, ZEISS, SUPRA, Jena, Germany), X-ray photoelectron spectroscopy (XPS, ESCALAB, 250Xi, Thermo Fisher, Waltham, MA, USA), transmission electron microscopy (TEM, JEOL, Tokyo, Japan), and Fourier transform infrared (FTIR, Bruker, Ettlingen, Germany) spectroscopy. The surface area was calculated using the Brunauer-Emmett-Teller (BET, ASAP 2020, Micromeritics, Atlanta, GA, USA).

The S/PAN/SiO_2_ and S/PAN electrodes were prepared by evenly grinding 80 wt % S/PAN/SiO_2_ (S/PAN) composites, 10 wt % Super P, and 10 wt % PVDF (50 mm diameter, Shanghai Xingya, Shanghai, China). The mixture was then dropwise added to NMP to yield a slurry, which was coated on nickel foam and dried in 60 °C oven for 12 h. the obtained platforms were then cut into circular electrodes. The graphite anode was comprised of graphite, Super P, and PVDF at the mass ratio of 90:3:7. A surface application technique was employed to apply SLMP suspended in xylene slurry onto the prefabricated graphite anodes. An SLMP: graphite molar ratio of 11:60 was utilized to compensate for the irreversible capacity and lithiating the graphite. Upon solvent evaporation, the anode sheets were calendered using manual rolling mill at 3 MPa. The cathode loading of each cell was 2.5 mg cm^−2^ and the graphite active material loading was about 3 mg cm^−2^.

The assembled 2025 button cell was completed in an argon-filled glove box. In half cells, a lithium chip was used as anode. In full cells, lithium was substituted by the lithiated graphite anode. The electrolyte was composed of 1M LiPF_6_ (Li zhiyuan, Shanghai, China) solution dissolved in ethyl carbonate (EC), dimethylcarbonate (DMC), and diethyl carbonate (DEC) at volume ratio of 1:1:1. Coin cells were assembled and pre-conditioned for 24 h at room temperature. The galvanostatic charge/discharge tests were conducted at different current densities (1 C = 1672 mA g^−1^) and voltage of 1.0–3.0 V Li^+^/Li.

## 3. Results and Discussion

**Figure 1:**
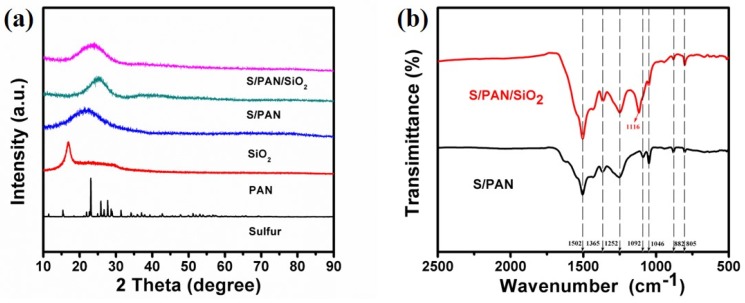
X-ray diffraction (XRD) and Fourier transform infrared (FTIR) images of S/PAN (S/PAN/SiO_2_) ternary composite.

Figure 1a represents the XRD patterns of S/PAN/SiO_2_ composite. The characteristic Fddd orthorhombic crystal structure peaks of elemental sulfur vanished from the XRD patterns of S/PAN composite. This could be due to trapping of S in the internal structure of the composite to form highly dispersed state, with crystalline sulfur and PAN most likely being converted to amorphous sulfurized PAN by heat-treatment [10,20]. Comparison of the characteristic bands between S/PAN/SiO_2_ and S/PAN composites revealed a broad peak at 23°, which can be indexed to mixed peaks of S/PAN and amorphous SiO_2_ [32,33]. The latter was probably induced by the dispersion of SiO_2_ on the S/PAN surface and a slight shift in characteristic peaks of the ternary composites to the left. It also shows that SiO_2_ and other components did not react during ball milling and heat treatment. Chemical analysis has shown that the sulfur content in the S/PAN/SiO_2_ ternary composite was 45 wt %.

In order to determine the various functional groups in the S/PAN/SiO_2_ composite, the FTIR analysis of the sample presents in Figure 1b. The characteristic peaks at 805, 1252, 1365 and 1502 cm^−1^ indicated the presence of C=C and C=N bonds in the composite [30]. The peaks at 882 cm^−1^ can be attributable to the S-S bonds, and those at 1046 and 1092 cm^−1^ can be assigned to the C-S stretching. The presence of the three peaks suggested that sulfur particles and PAN formed sulfurized-polyacrylonitrile [34]. The peak at 1116 cm^−1^ was associated with the asymmetric stretching vibration of Si-O-Si in SiO_2_ [33].

**Figure 2:**
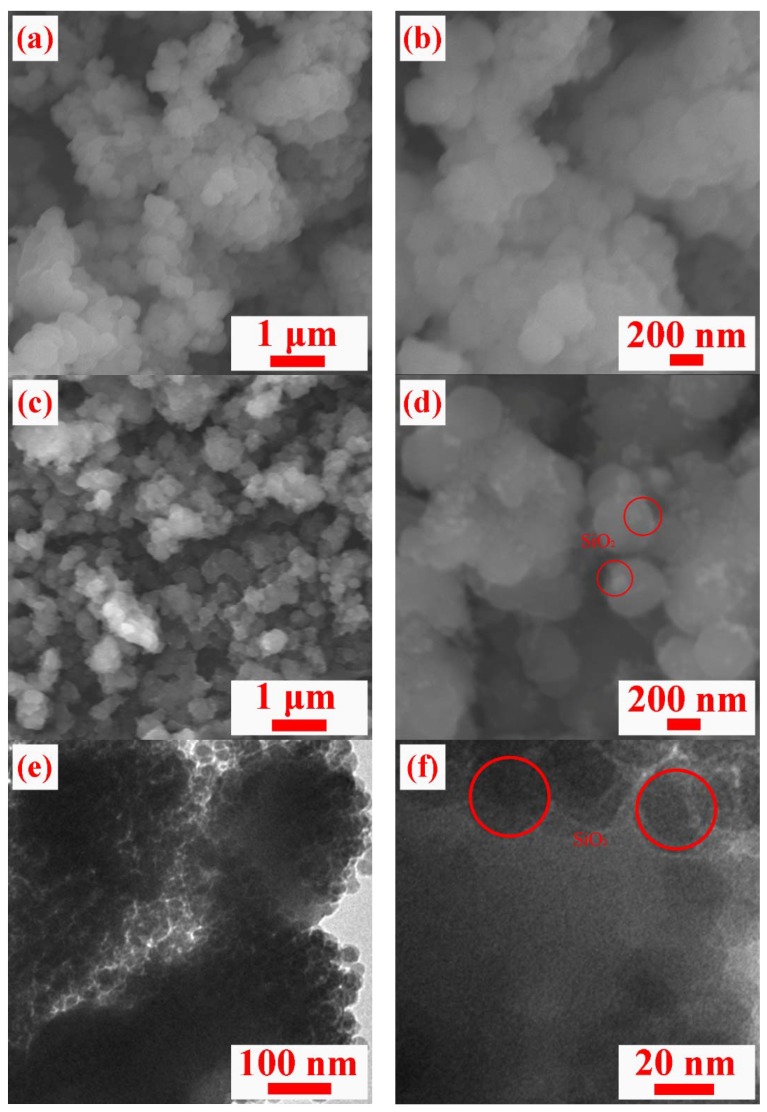
SEM images (**a**,**b**) of S/PAN composite, SEM (**c**,**d**) and TEM images (**e**,**f**) of S/PAN/SiO_2_ ternary composites.

The microscopic morphologies of S/PAN/SiO_2_ composites were revealed by SEM. The surface morphology of S/PAN binary composite underwent remarkable changes in the presence of small amounts of SiO_2_ nanoparticles. The typical S/PAN nanostructure depicted in Figure 2a was composed numerous agglomerated particles. The S/PAN composite showed a bulk structure with very compact particles and smooth surface (Figure 2b). By comparison, the S/PAN/SiO_2_ ternary composite consisted of nanosized primary particles (Figure 2c,d), resulting in a rough surface of the ternary composite. The BET specific surface area of S/PAN/SiO_2_ composite was estimated to 33.75 m^2^ g^−1^, which was significantly higher than those of S/PAN binary composites that were published previously [20,31]. Therefore, the surface area was increased significantly by adding the additive with a nano structure. This should facilitate the contact between the electrolyte and electrodes. Moreover, the ternary composite contained many nano-sized particles, creating a three-dimensional (3D) porous nanostructure, it is beneficial to ion diffusion in Li/S battery and the SiO_2_ suppresses the separation and agglomeration of active materials in the composite. The TEM image of S/PAN/SiO_2_ composite were shown in Figure 2e,f, the “dark dots” were the amorphous SiO_2_ particles well-dispersed in the composite bulk, which agrees well with the wide peaks in the XRD patterns. The as-prepared ternary composite was enabled to maintain the homogeneous distribution of its components and unchanged morphology during discharge-charge cycling, and retain the reactive sites in its nanosized structure [35].

**Figure 3:**
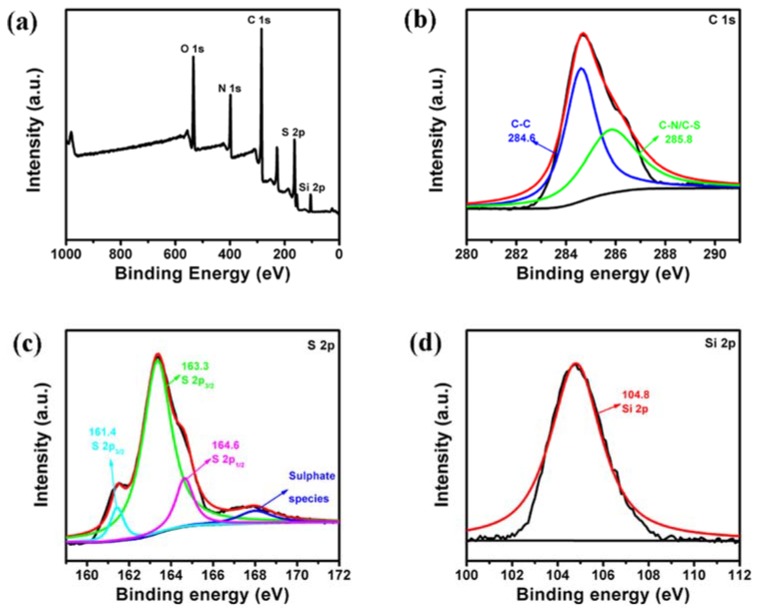
(**a**) Survey X-ray photoelectron spectroscopy (XPS) spectrum of S/PAN/SiO_2_ composite, (**b**) C 1s, (**c**) S 2p and (**d**) Si 2p spectra of S/PAN/SiO_2_ composite.

To further determine the chemical bonds in the S/PAN/SiO_2_ composite, the XPS analysis of the sample is shown in Figure 3a–d. The C 1s, S 2p, and Si 2p peaks were all detected in the spectra. Figure 3b revealed the presence of a high-resolution peak of C 1s, as well as two peaks corresponding to C-C (284.6 eV) and C-N/C-S. The XPS C 1s spectra of S/PAN/SiO_2_ composite confirmed the presence of a distinct peak at 285.8 eV, corresponding to the C-S/C-N bonds. This indicated the existence of certain chemical bonding between S and PAN in the S/PAN/SiO_2_ composite. Figure 3c reveals the high-resolution S 2p peak. The S 2p_3/2_ peak located at 161.4 and 163.3 eV were associated with C-S bond, attributed to single C-S bond and C-S bond in short-chain sulfide. The S 2p_1/2_ peak at 164.6 eV was assigned to the S-S bond [21]. In XPS spectrum of Si 2p (Figure 3d), the peak at 104.8 eV was also present in the characteristic peak of SiO_2_ [36]. These data were in accordance with the XRD patterns.

**Figure 4:**
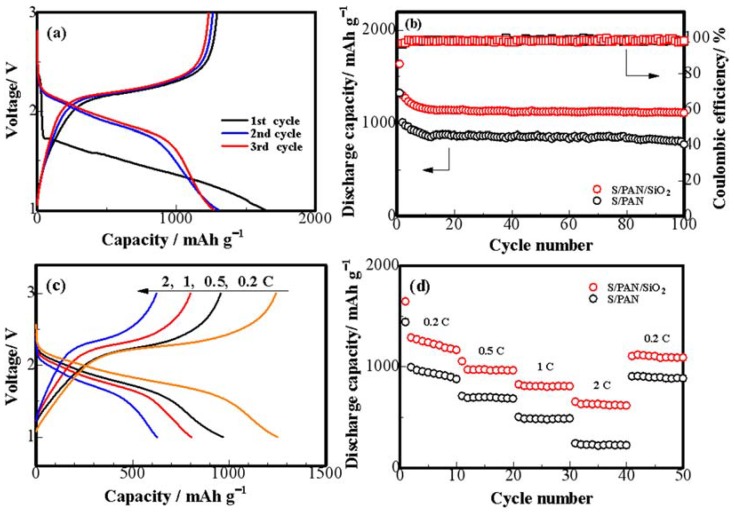
(**a**) Discharge/charge performance of S/PAN/SiO_2_ half-cell at 0.2 C between 1 V and 3 V. (**b**) Cycling performance and coulombic efficiency of S/PAN/SiO_2_ half-cell at 0.2 C. (**c**) Rate performance of S/PAN/SiO_2_ cell at various current densities. (**d**) Rate performance of S/PAN/SiO_2_ and S/PAN half-cell at various current densities.

To figure out the electrochemical performances, the Li/S half cells were assembled while using lithium metal as anode, S/PAN/SiO_2_ composites as cathode, and 1M LiPF_6_ as electrolyte. Figure 4a shows the discharge/charge curves of S/PAN/SiO_2_ half batteries. A method of containment at the cathode is to provide additives in the cathode matrix that can attract and hold polysulfides so that they do not diffuse to the negative electrode. We utilized SiO_2_ as an additive to S/PAN electrode. The main interaction that the polysulfides have with the additive is through surface sorption, and therefore the surface area is increased significantly by synthesizing the additive with a nano structure. the SiO_2_ additive was able to sorb polysulfides during the intermediate discharge and release them near the end of discharge so that they could be further reduced in the S/PAN matrix with most of the sulfur being reversibly sorbed in the SiO_2_. The kinetics improvement and the polarization decrease achieved in the system by the addition of SiO_2_ could be very beneficial for the utilization of the low-conductive sulfur active material in the composite cathode, and consequently improved the energy and power density of the battery [20,30]. The initial discharge curve consisted of a rapid drop curve around 2.4 V and flat discharge curve around 1.6 V. The initial charge-discharge process was typical of lithium-sulfur battery reactions [10]. During the following two cycles, the discharge curve was mainly composed of two lines with different inclinations. The first part of the curve looked relatively flat (from 2.2 V to 1.6 V) and the second was steep (from 1.6 V to 1.0 V). The two curves indicated the available capacity of about 1266 mAh g^−1^. Figure 4b confirmed a coulombic efficiency of binary and ternary composite electrode half cells close to 100%. However, the S/PAN/SiO_2_ half-cell showed a higher specific discharge capacity, which can be attributed to added nanosized SiO_2_. Moreover, the S/PAN/SiO_2_ electrode maintained a discharge capacity of 1106 mAh g^−1^ at 0.2 C after 100 cycles. The S/PAN/SiO_2_ half-cell exhibited specific discharge capacities of 1268, 969, 806, and 622 mAh g^−1^ at current densities of 0.2, 0.5, 1 and 2 C, respectively (Figure 4c). By comparison, the S/PAN half-cell was also tested under the same conditions and the data are gathered in Figure 4d. Obviously, the cell with the S/PAN/SiO_2_ composite cathode showed an enhanced rate capability. This is, again, due to the significant improvement of the charge transfer properties of the composite cathode and its stability by the SiO_2_ additive that was observed in this work [37].

**Figure 5:**
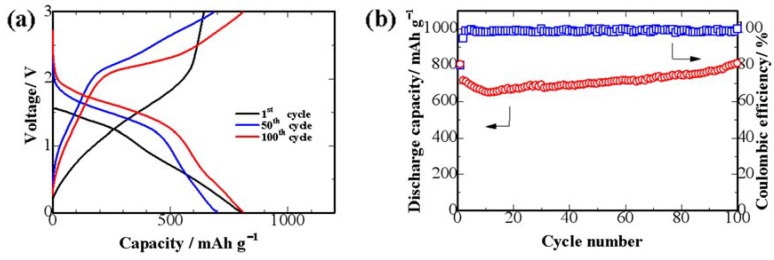
(**a**) Discharge/charge performance of S/PAN/SiO_2_ full-cell at 0.2 C between 0.1 V and 3 V. (**b**) Cycling performance and coulombic efficiency of S/PAN/SiO_2_ full-cell at 0.2 C.

As shown in Figure 5, the prelithiated graphite//S/PAN/SiO_2_ composite system exhibited an initial capacity of 804 mAh g^−1^ and specific capacity of 810 mAh g^−1^ after 100th cycle. Hence, extremely low capacity decay rate was registered. Figure 5a depicts the coulomb efficiency of the full cell, which was close to 100%. The cycling curve of the full cell indicated a slight decrease from the initial cycle up to the 10th cycle then slowly increased up to 100th cycle. Although the amorphous S/PAN can provide good mechanical support for SiO_2_ spheres, the structural stability cannot be guaranteed due to repeated volume changes during cycling. This led to decrease in capacity. Meanwhile, the added amorphous nano-sized SiO_2_ absorbed more electrolyte and polysulfide on the surface of rough ternary composite. The discharge capacity also gradually increased in subsequent cycles, which is may due to that the pre-lithiated graphite was not fully lithiated, meaning that the electrode underwent slow activation. When the electrode was in contact with the electrolyte, the SLMP in the anode released lithium ions, making it slow to activate. As the battery cycles increases, the anode side of the graphite was completely lithiated, and the battery exhibited a gradual increase in discharge specific capacity [28,29,38].

When compared to other related work (Table 1), our S/PAN/SiO_2_ composite exhibited superior electrochemical performance.

## 4. Conclusions

Sulfur/lithium-ion full batteries were assembled while using ternary S/PAN/SiO_2_ composite as cathode and a prelithiated graphite as anode. SLMP was successfully applied to lithiation of graphite anode. Nano-sized SiO_2_ was found to be uniformly distributed on S/PAN composites, which served as an effective additive due to its ultra high absorbtion ability and enhanced trapping soluble polysulfide. The S/PAN/SiO_2_ composite cathode half-cell showed a high discharge capacity of 1106 mAh g^−1^ after 100 cycles at 0.2 C. The pre-lithiated graphite//S/PAN/SiO_2_ composite full cell system delivered a specific capacity of 810 mAh g^−1^ over 100 cycles. These findings look promising for future use in energy conversion and storage devices.

## Figures and Tables

**Table 1:** Literature comparison of the electrochemical performances of cathode materials for lithium sulfur batteries.

Cathodes	Sulfur Loading (wt %)	Current Density	Initial Discharge Capacity (mAh/g)	Discharge Capacity (mAh/g) (After n^th^ Cycle)	References
S/DPAN	48	0.2 C	1550	1050 (80)	[10]
S/PAN/Mg_0.6_Ni_0.4_O	38.5	0.1 C	1540	1200 (100)	[20]
S@pPAN	37.64	200 mA/g	2200	1700 (100)	[32]
S@pPAN	40.9	0.5 C	1510	1100 (100)	[38]
S/PAN/Graphene	47.3	0.1 C	719	612 (10)	[39]
S@pPAN // Prelithiated SiO_x_/C	87	0.36 C	850	600 (100)	[40]
MesoC/Sulfur // Prelithiated Graphite	-	0.1 C	608	405 (105)	[41]
S/PAN/SiO_2_ // Prelithiated Graphite	45	0.2 C	804	810 (100)	This work

## References

[1] Armand M., Tarascon J.-M. (2008). Building better batteries. Nature.

[2] Wei Z.B., Meng S.J., Xiong B.Y., Ji D.X., JetTseng K. (2016). Enhanced online model identification and state of charge estimation for lithium-ion battery with a FBCRLS based observer. Appl. Energy.

[3] Li H.L., Wei Y.Q., Zhang Y.G., Zhang C.W., Wang G.K., Zhao Y., Yin F.X., Bakenov Z. (2016). In situ sol-gel synthesis of ultrafine ZnO nanocrystals anchored on graphene as anode material for lithium-ion batteries. Ceram. Int..

[4] Wei Z.B., Zhao J.Y., Ji D.X., JetTseng K. (2017). A multi-timescale estimator for battery state of charge and capacity dual estimation based on an online identified model. Appl. Energy.

[5] Zhang Y.G., Li Y., Li H.P., Yin F.X., Zhao Y., Bakenov Z. (2016). Synthesis of hierarchical MoS2 microspheres composed of nanosheets assembled via facile hydrothermal method as anode material for lithium-ion batteries. J. Nanopart. Res..

[6] Wei Z.B., Bhattarai A., Zou C.F., Meng S.J., Lim T.M., Skyllas-Kazacos M. (2018). Real-time monitoring of capacity loss for vanadium redox flow battery. J. Power Sources.

[7] Kumaresan K., Mikhaylik Y., White R.E. (2008). A Mathematical Model for a Lithium-Sulfur Cell. J. Electrochem. Soc..

[8] Doan T.N.L., Ghaznavi M., Konarov A., Zhang Y.G., Chen P. (2014). Cyclability of sulfur/dehydrogenated polyacrylonitrile composite cathode in lithium–sulfur batteries. J. Solid State Electr..

[9] Grixti S., Mukherjee S., Singh C.V. (2018). Two-Dimensional Boron as an impressive Lithium-Sulphur battery cathode material. Energy Storage Mater..

[10] Konarov A., Gosselink D., Doan T.N.L., Zhang Y.G., Zhao Y., Chen P. (2014). Simple, scalable, and economical preparation of sulfur-PAN composite cathodes for Li/S batteries. J. Power Sources.

[11] Li G., Wang X.L., Seo M.H., Li M., Ma L., Yuan Y.F., Wu T.P., Yu A.P., Wang S., Lu J. (2018). Chemisorption of polysulfides through redox reactions with organic molecules for lithium-sulfur batteries. Nat. Commun..

[12] Anilkumar K.M., Jinisha B., Manoj M., Pradeep V.S., Jayalekshmi S. (2018). Layered sulfur/PEDOT: PSS nano composite electrodes for lithium sulfur cell applications. Appl. Surf. Sci..

[13] Yuan G.H., Yin F.X., Zhao Y., Bakenov Z., Wang G.K., Zhang Y.G. (2016). Corn stalk-derived activated carbon with a stacking sheet-like structure as sulfur cathode supporter for lithium/sulfur batteries. Ionics.

[14] HyunKim P.J., Kim K., Pol V.G. (2018). Towards highly stable lithium sulfur batteries: Surface functionalization of carbon nanotube scaffolds. Carbon.

[15] Scholz J., Kayaalp B., Juhl A.C., Clemens D., Fröba M., Mascotto S. (2018). Severe Loss of Confined Sulfur in Nano porous Carbon for Li-S Batteries under Wetting Conditions. ACS Energy Lett..

[16] Zhang Y.G., Sun L.C., Li H.P., Tan T.Z., Li J.D. (2018). Porous three-dimensional reduced graphene oxide for high-performance lithium-sulfur batteries. J. Alloys Compd..

[17] Arava L.M.R., Gopalakrishnan D., Lee A. (2018). Electrocatalytically Active Niobium Sulfide Modified Carbon Cloth for Lithium-Sulfur Batteries. J. Electrochem. Energy.

[18] Tian Y., Sun Z.H., Zhang Y.G., Wang X., Bakenov Z., Yin F.X. (2018). Micro-Spherical Sulfur/Graphene Oxide Composite via Spray Drying for High Performance Lithium Sulfur Batteries. Nanomaterials.

[19] Jeong T.-G., Lee Y.-S., Cho B.W., Kim Y.-T., Jung H.-G., Chung K.Y. (2018). Improved performance of dual-conducting polymer-coated sulfur composite with high sulfur utilization for lithium-sulfur batteries. J. Alloys Compd..

[20] Zhang Y.G., Zhao Y., Yermukhambetova A., Bakenov Z., Chen P. (2013). Ternary sulfur/polyacrylonitrile/Mg0.6Ni0.4O composite cathodes for high performance lithium/sulfur batteries. J. Mater. Chem. A.

[21] Li F., Kaiser M.R., Ma J., Guo Z.P., Liu H.K., Wang J.Z. (2018). Free-standing sulfur-polypyrrole cathode in conjunction with polypyrrole-coated separator for flexible Li-S batteries. Energy Storage Mater..

[22] Yin F.X., Liu X.Y., Zhang Y.G., Zhao Y., Menbayeva A., Bakenovd Z., Wang X. (2017). Well-dispersed sulfur anchored on interconnected polypyrrole nanofiber network as high performance cathode for lithium-sulfur batteries. Solid State Sci..

[23] Lee F., Tsaia M.-C., Lina M.-H., Nimaha Y.L., Hya S., Kuoa C.Y., Chenga J.-H., Ricka J., Sub W.-N., Hwanga B.-J. (2017). Capacity retention of lithium sulfur batteries enhanced with nano-sized TiO_2_-embedded polyethylene oxide. J. Mater. Chem. A.

[24] Wang J.L., Yang J., Wan C.R., Du K., Xie J.Y., Xu N.X. (2003). Sulfur Composite Cathode Materials for Rechargeable Lithium Batteries. Adv. Funct. Mater..

[25] Yermukhambetova A., Bakenov Z., Zhang Y.G., Darr J.A., Brett D.J.L., Shearing P.R. (2016). Examining the effect of nanosized Mg_0.6_Ni_0.4_O and Al_2_O_3_ additives on S/polyaniline cathodes for lithium–sulphur batteries. J. Eelctroanal. Chem..

[26] Zhang Y.G., Bakenov Z., Zhao Y., Konarov A., Doan T.N.L., Sun K.E.K., Yermukhambetova A., Chen P. (2013). Effect of nanosized Mg_0.6_Ni_0.4_O prepared by self-propagating high temperature synthesis on sulfur cathode performance in Li/S batteries. Powder Technol..

[27] Agostini M., Scrosati B., Hassoun J. (2015). An Advanced Lithium-Ion Sulfur Battery for High Energy Storage. Adv. Eng. Mater..

[28] Wang L., Fu Y.B., Battaglia V.S., Liu G. (2013). SBR-PVDF based binder for the application of SLMP in graphite anodes. Rsc. Adv..

[29] Wang Z.H., Fu Y.B., Zhang Z.C., Yuan S.W., Amine K., Battaglia V., Liu G. (2014). Application of Stabilized Lithium Metal Powder (SLMP^®^) in graphite anode—A high efficient prelithiation method for lithium-ion batteries. J. Power Sources.

[30] Evers S., Nazar L.F. (2012). New approaches for high energy density lithium–sulfur battery cathodes. Acc. Chem. Res..

[31] Zhao Y., Zhang X.M., He Y.S., Liu N., Tan T.Z., Liang C.Y. (2017). Biomass Derived Nitrogen-Doped Highly Porous Carbon Material with a Hierarchical Porous Structure for High-Performance Lithium/Sulfur Batteries. Materials.

[32] Jin Z.Q., Liu Y.G., Wang W.K., Wang A.B., Hu B.W., Shen M., Gao T., Zhao P.C., Yang Y.S. (2018). A new insight into the lithium storage mechanism of sulfurized polyacrylonitrile with no soluble intermediates. Energy Storage Mater..

[33] Xia H.Y., Yin Z.C., Zheng F.C., Zhang Y.G. (2017). Facile synthesis of SiO2/C composites as anode materials for lithium-ion batteries. Mater. Lett..

[34] Fanous J., Wegner M., Grimminger J., Andresen A., Buchmeiser M.R. (2011). Structure-Related Electrochemistry of Sulfur-Poly (acrylonitrile) Composite Cathode Materials for Rechargeable Lithium Batteries. Chem. Mater..

[35] Jiang Y., Mu D.B., Chen S., Wu B.R., Zhao Z.K., Wu Y.Z., Ding Z.P., Wu F. (2018). Hollow silica spheres with facile carbon modification as an anode material for lithium-ion batteries. J. Alloys Compd..

[36] Back C.K., Kim T.J., Choi N.S. (2014). Activated natural porous silicate for a highly promising SiO_x_ nanostructure finely impregnated with carbon nanofiber as a high performance anode material for lithium-ion batteries. J. Mater. Chem. A.

[37] Jiao M.L., Liu K.L., Shi Z.Q., Wang C.Y. (2016). SiO*_2_*/carbon composite microspheres with hollow core-shell structure as a high stability electrode for lithium ion batteries. Chemelectrochem.

[38] Zhang Y.G., Zhao Y., Bakenov Z., Konarov A., Chen P. (2014). Preparation of novel network nanostructured sulfur composite cathode with enhanced stable cycle performance. J. Power Sources.

[39] Zhang Y.G., Zhao Y., Bakenov Z., Babaa M.-R., Konarov A., Ding C., Chen P. (2013). Effect of graphene on sulfur/polyacrylonitrile nanocomposite cathode in high performance lithium/sulfur batteries. J. Electrochem. Soc..

[40] Shi L., Liu Y.G., Wang W.K., Wang A.B., Jin Z.Q., Wu F., Yang Y.S. (2017). High-safety lithium-ion sulfur battery with sulfurized polyacrylonitrile cathode, prelithiated SiO_x_/C anode and carbonate-based electrolyte. J. Alloys Compd..

[41] Zeng P., Han Y.M., Duan X.B., Jia G.C., Huang L.W., Chen Y.G. (2017). A stable graphite electrode in superconcentrated LiTFSI-DME/DOL electrolyte and its application in lithium-sulfur full battery. Mater. Res. Bull..

